# Controlled gelation kinetics of cucurbit[7]uril-adamantane cross-linked supramolecular hydrogels with competing guest molecules

**DOI:** 10.1038/srep20722

**Published:** 2016-02-05

**Authors:** Hao Chen, Shengzhen Hou, Haili Ma, Xu Li, Yebang Tan

**Affiliations:** 1 School of Chemistry and Chemical Engineering, Shandong University, Jinan 250100, People’s Republic of China; 2 The Key Laboratory of Special Functional Aggregated Materials, Ministry of Education, Shandong University, Jinan 250100, People’s Republic of China; 3 Institute of Materials Research and Engineering, 3 Research Link, Singapore 117602

## Abstract

Gelation kinetics of hydrogels is closely linked to many applications such as the development of injectable and printable hydrogels. However, the control of gelation kinetics without compromising the structure and other properties of the hydrogels, remains a challenge. Here, we demonstrate a method to control the gelation kinetics of cucurbit[7]uril-adamantane (CB[7]-AD) cross-linked supramolecular hydrogels by using competing guest molecules. The association between CB[7] and AD moieties on the polymer backbone was impeded by pre-occupying the CB[7] cavity with competing guest molecules. By using various guest molecules and concentrations, the gelation of the hydrogels could be varied from seconds to hours. The strong interaction of CB[7]-AD pair endue the hydrogels good mechanical properties and stability. Moreover, the binding of functionalized guest molecules of CB[7] moieties offers a facile approach for tailoring of the hydrogels’ scaffold. Combined with hydrogel injection and printing technology, this method offers an approach for the development of hydrogels with advanced temporal and spatial complexity.

The development of gelation process with desired control is a major goal of hydrogel technology. Many applications such as the development of injectable and printable hydrogels are closely linked to this characteristic^1^,^2^,^3^. In order to meet this goal, gelation mechanisms that are based on physical changes including temperature^4^,^5^, ionic strength^6^,^7^, pH value^8^, shear force^9^, and chemical reactions such as the Schiff base formation^10^,^11^, disulfide bond formation^12^, Michael addition^13^ and photopolymerization^14^,^15^,^16^ have been explored^17^. However, it remains a challenge to have effective control over gelation kinetics without compromising the structure and other properties of hydrogels. Although the gelation of hydrogels based on physical changes is triggered by external stimuli, their gelation usually proceeds through a sharp transition when the corresponding external stimuli are applied^4^,^5^,^6^,^7^,^8^. Control over the gelation kinetics of hydrogels based on chemical reactions is plausible. The gelation kinetics varies based on the composition of gelators. Factors including the concentration and the functionalization degree of polymer precursors and the concentration of cross-linkers determine the gelation kinetics. However, the structure and properties of hydrogels (e.g. mechanical strength) will change following the change of composition^18^,^19^. Combined with the trigger of light, photopolymerization is able to offer controllable gelation kinetics of hydrogels; although its application is limited to the availability of UV light^14^,^15^,^16^. To date, there is unmet need for gelation mechanisms with controllable gelation kinetics which are independent of other properties.

The transient inherence of non-covalent interactions offers possibility in controlling the gelation kinetics of supramolecular polymeric hydrogels from the molecular scale. The fast association of non-covalently complementary pairs can be impeded by pre-saturation of one moiety with weaker competing molecules^20^,^21^,^22^,^23^,^24^. The additional dissociation process of the weaker complementary pair before the formation of the desired complementary pair is attributed to this impedance^20^,^21^,^22^,^23^. The degree of the slowing is determined by the competing molecules and their concentrations. Based on the control of non-covalent interactions, the gelation kinetics of supramolecular hydrogels with corresponding non-covalent crosslinks could also be controllable. Moreover, the non-covalent moieties in the hydrogels can also be used for the binding of complementary moieties with functional groups, which enhance the tailorability of the supramolecular hydrogels^25^,^26^.

Cucurbiturils (CBs) are ideal host molecules to demonstrate the idea that control the gelation kinetics of supramolecular hydrogels using competing guest molecules. Consisting of methylene bridged glycolurils, these pumpkin shaped, rigid molecules demonstrate amazing binding affinity and selectivity to a wide range of guest molecules^27^,^28^,^29^. With such binding properties, numerous competing guest molecules can be employed in the control of certain CB based host-guest interactions, which offer a wide range of interaction dynamics. Herein, we selected the cucurbit[7]uril-adamantane (CB[7]-AD) complementary pair as the non-covalent crosslinks, with a binding affinity estimated up to 10^14^ M^−1^ 24(Fig. 1). Thus we synthesized two *N,N*-dimethyl acrylamide based polymers with CB[7] and AD pendants^30^. Through pre-saturation the CB[7] pendent polymer with various competing guest molecules and concentrations, the gelation kinetics of the two polymers changes significantly and the gelation time varies from seconds to hours. The strong interaction of the CB[7]-AD pair endue the supramolecular hydrogels unique mechanical properties such as high elasticity, good stability and shape persistence that are rarely observed for supramolecular hydrogels. Furthermore, the supramolecular inherence allows the hydrogels easily to be decorated with functional groups through host-guest interactions. Based on these features, the hydrogels were demonstrated applicable for both of the injection and printing operation with good strength and tailorable properties.

## Results

### Gelation of the CB[7]-AD cross-linked supramolecular hydrogels

**Figure 2:**
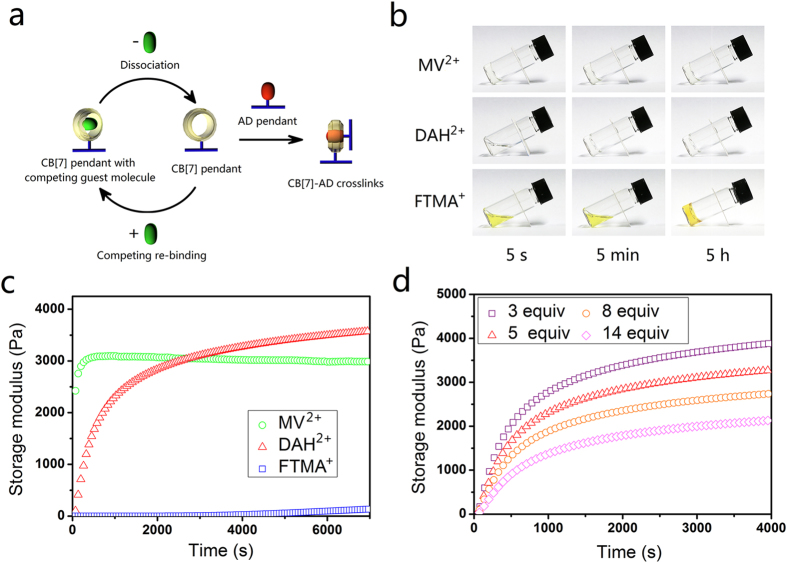
Control gelation kinetics of the hydrogels through varying competing guest molecules and their concentrations. (**a**) Formation process of CB[7]-AD crosslinks that treated with competing guest molecules. Competing guest molecules are released from CB[7] cavity before the formation of the CB[7]-AD crosslinks. Competing guest molecules may also re-binding the free CB[7] cavity. The dissociation dynamics of competing guests is related to the release process. And the association dynamics and their concentration are related to the re-binding process. (**b**) Vial inclination observation and (**c**) oscillatory rheological experiments of the hydrogels treated with different competing guest molecules. The gelation kinetics changed significantly through varying the competing guest molecules (5 equiv competing guest molecules *versus* CB[7] pendants was used). (**d**) Oscillatory rheological experiments of the hydrogels treated with different concentration of DAH^2+^. The gelation kinetics changed mild through varying the concentration of competing guest molecules. All the experiments were performed with polymer content of 2 wt% with CB[7]-AD at the stoichiometric point (1:1 in mole).

Due to the fast association between CB[7] and AD pendants, directly mixing of the CB[7] pendent polymer and AD pendent polymer by vortex mixing gave aggregates rather than integral hydrogel (Supplementary Fig. S1). Through treat the CB[7] pendant polymer with competing guest molecules before mix the polymers, the association of CB[7] and AD pendants is sufficiently decelerated. And integral hydrogels could form (Fig. 2). Based on the molecular mechanism illustrated in Fig. 2a, the dynamic properties of competing guest molecules, which are determined by the sort of them, have an effect on the gelation process of the hydrogels. Besides, the concentration of the competing guest molecules also affects the gelation process since it is related to the competing re-binding process.

The gelation processes of the hydrogels that treated with dimethyl viologen diiodide (MV^2+^), 1,6-diaminohexane dihydrochloride (DAH^2+^) and (ferrocenylmethyl)trimethyl ammonium iodide (FTMA^+^) were characterized by using vial inclination experiments (Fig. 2b) and oscillatory rheological experiments (Fig. 2c). The hydrogel that treated with MV^2+^ demonstrated fastest gelation with among the three hydrogels. It gelled within less than 5 seconds. The gelation started before the polymer mixture was loaded on the geometry, and the hydrogel structure may be partially damaged during the loading process. Based on the obtained data, the gelation was estimated to have finished after several minutes. The hydrogel that treated with DAH^2+^ gelled with a slower rate. The polymer mixture was able to flow during the first minute with increasing in viscosity. Its storage modulus increased drastically during first half an hour before decelerating. The hydrogel that treated with FTMA^+^ displayed the slowest gelation kinetics. The mixed solution flowed like a liquid for the first two hours and formed a hydrogel within several more hours. Its storage modulus stayed at a low level and kept increasing even after 12 h (Supplementary Fig. S2). In fact, the gelation process was shown to last for several days (*vide infra*).

The effect of the concentration of competing guest molecules is illustrated in Fig. 2d. DAH^2+^ was selected as the model competing guest molecules in this test as it offers proper gelation kinetics that would facilitate the experiments. The gelation kinetics decelerated with the increasing concentration of competing guest molecules, as expected. Compared with using different guest molecules, the change of the concentration of the competing guest molecules has a much milder effect on the gelation kinetics.

### Mechanism study of hydrogel gelation

**Figure 3:**
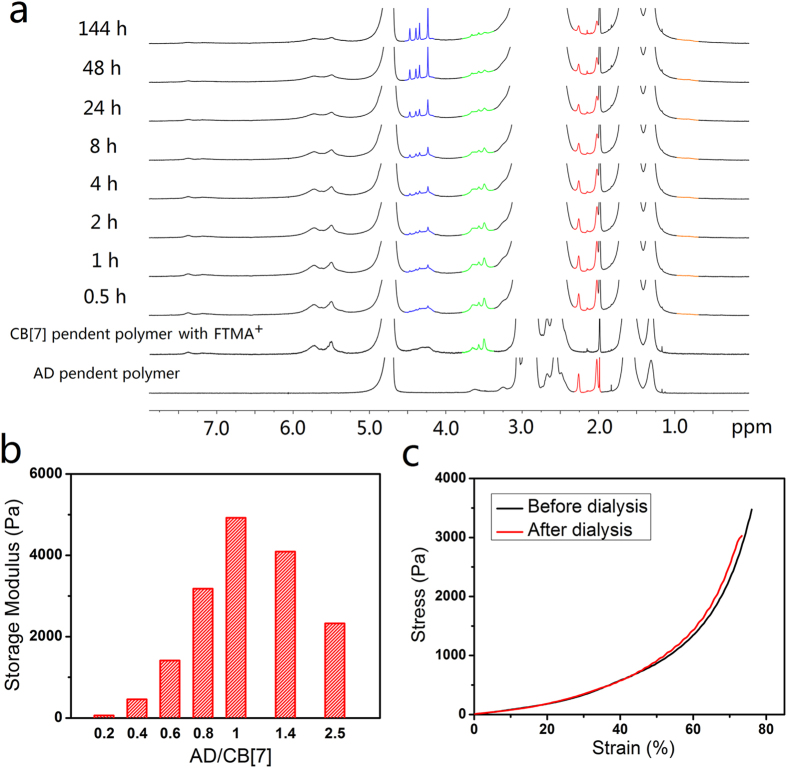
Mechanism study of hydrogel gelation. (**a**) NMR monitoring of the gelation process of the hydrogel treated with 1 equiv FTMA^+^ (Blue for signals corresponding to protons of free FTMA^+^, green for protons of bounded FTMA^+^, red for protons of unbounded AD moiety and orange for protons of bounded AD moiety). FTMA^+^ was released from CB[7] cavity accompanied by the formation of CB[7]-AD crosslinks. (**b**) Storage moduli of the hydrogels with different CB[7]/AD stoichiometry (3 equiv DAH^2+^ was used). The hydrogels demonstrated maximum storage modulus with CB[7]-AD at the stoichiometric point (1:1 in mole). (**c**) Compression experiments of the hydrogel before and after the removing of competing guest molecule (3 equiv FTMA^+^ was used with CB[7]-AD at the stoichiometric point during sample preparation). The competing guest molecule shows negligible effect on the mechanical property of the hydrogels.

NMR method was firstly used in monitoring the gelation process of the hydrogels (Fig. 3a). The signals of protons on guest molecules demonstrates significant chemical shift when the guest molecules were encapsulated inside CB cavity^31^ (Supplementary Fig. S3). By this manner we can have a view of the gelation process from molecular scale. Due to the slow gelation kinetics of the FTMA^+^ treated hydrogels, they were employed for the NMR experiments to facilitate the observation. Only 1 equiv (*versus* CB[7] moiety) FTMA^+^ was used in order to minimize the interference for the observation of changing on peaks. Upon the mixing of the two polymers, peaks at around 3.5 ppm which represent the bounded FTMA^+^ began to diminish followed by the appearance of sharp peaks at around 4.3 ppm, clearly showing the release of FTMA^+^ from CB[7] cavity during the gelation process. Meanwhile, peaks of AD moiety at 2.0 and 2.3 ppm also diminished accompanied by the appearance of a new peak at 0.8 ppm, indicating that AD moieties were being encapsulated by CB[7] moieties. During the gelation process, the peaks of the polymers broadened, which matches with the fact that gelation impedes the mobility of polymer chain. Although the majority of the supramolecular interactions finished within 48 h, it was difficult for them to undergo full complexation even after 6 days. This phenomenon may be attributed to the low concentration of the active moieties as well as their restricted diffusion induced by the cross-linked polymer network.

Supramolecular polymeric hydrogels usually demonstrate optimal mechanical strength with the complementary pairs at stoichiometric point as it offers the highest cross-link density^32^,^33^. The storage moduli of hydrogels with various compositions and fixed solid contents (2 wt%) were summarized in Fig. 3b. The hydrogels demonstrate a highest G’ with the AD/CB[7] ratio equal to the stoichiometric point. Either excess amount of AD moiety or CB[7] moiety decrease the hydrogels’ G’. This result also supports that the CB[7]-AD pair works as supramolecular crosslinks.

The role of the competing guest molecules in the hydrogels was also assessed. Compression tests of the hydrogels before and after removing competing guest molecules were performed (Fig. 3c). The hydrogels with and without FTMA^+^ perform similar in the compression tests as the strain-stress curves of the hydrogels almost overlapped, indicating that the competing guest molecules are not a structural factor of hydrogels and have little effect on the mechanical property of them.

### Mechanical properties of the hydrogels

**Figure 4:**
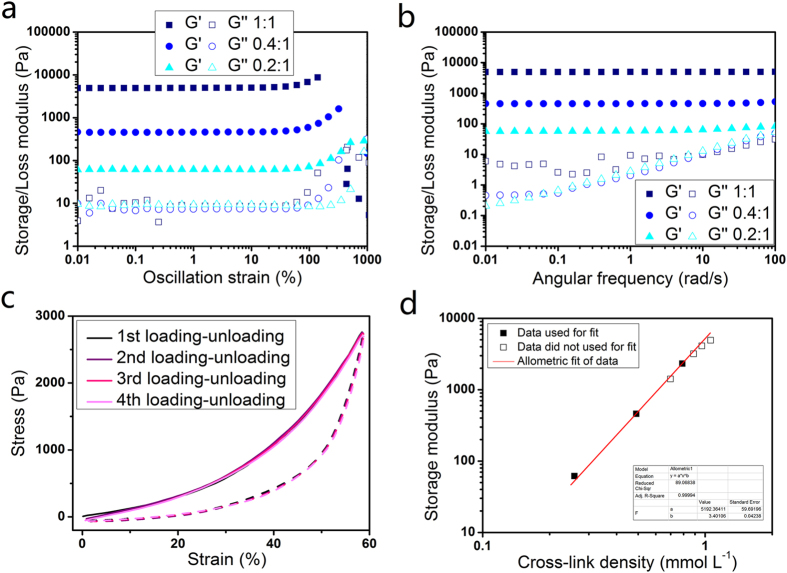
Mechanical properties of the hydrogels. Oscillatory rheological experiments of (**a**) amplitude sweep and (**b**) frequency sweep of the hydrogels. (**c**) Compression-decompression cycles of the hydrogels with CB[7]/AD ratio at stoichiometric point. (**d**) Plot of storage modulus *versus* cross-link density. All the experiments were performed with solid content of 2 wt% of the hydrogels that treated with 3 equiv DAH^2+^.

Figure 4a depicts the storage and loss moduli *versus* strain of the hydrogels obtained from oscillation amplitude sweep. The hydrogels demonstrate high mechanical strength, elasticity and high flexibility. The storage modulus of the hydrogel with CB[7]-AD at stoichiometric point is 5 kPa. This value is amazingly high for supramolecular hydrogels especially take its high water content (98 wt%) into consideration. Although for other supramolecular hydrogels, higher storage moduli were observed. However a much lower water content (less than 90 wt%) and much higher cross-link density are needed^33^. The storage modulus of the hydrogels is much high than their loss modulus, showing that the hydrogels are highly elastic. For the hydrogel with 1:1 AD-CB[7] complementary moieties, the storage modulus is nearly 3 orders of magnitude higher than the loss modulus, which means over 99% energy is stored in the hydrogel network as elastic potential energy under deformation. It is interesting to find the hydrogels with different composition demonstrated the similar loss modulus. Based on the fact that they have the same polymer content, the energy loss is suggested only dissipated by the disentanglement of the polymer chain rather than the breaking of CB[7]-AD crosslinks. The hydrogels have similar linear viscoselastic regions up to 60% of strain with various compositions. The moduli of the hydrogels increase with strain exceed the linear viscoselastic region, indicating the hydrogels are strain hardening^34^. The break-down strain varies based the composition of hydrogels. All of the hydrogels demonstrated break-down strain higher than 100%. Lower cross-link density endue the hydrogels higher flexibility - the hydrogel formed by 1:5 AD/CB[7] has a break-down strain over 1000%, which is rarely found for supramolecular hydrogels.

Oscillation frequency sweeps of the hydrogels were performed at 2% strain based on the obtained linear viscoselastic region of the hydrogles (Fig. 4b). There is not even a trend of cross-section found in the low frequency region of the storage modulus and loss modulus, indicating that the relaxation rate of the hydrogels is extremely slow. Cyclic compression test of the hydrogel further confirms the low relaxation rate of the hydrogel (Fig. 4c). The compression and de-compression curves of the hydrogel overlapped in high precision within 4 cycles with maximum stress and strain up to 2760 kPa and 58% respectively. The strain and stress are relatively high for the hydrogel as its break-down stress and strain are 4739 kPa and 71% respectively (Supplementary Fig. S4). As applied stress can change the equilibrium of non-covalent interaction, it is quite amazing for the hydrogel to display such good shape persistence without any relaxation under considerable high pressure.

The data of storage modulus *versus* cross-link density was summarized in Fig. 4d. It was found that the data can be well fitted within a straight line with a double logarithmic coordinate system. Due to the fact that it is hard to reach the final equilibrium for hydrogels when the ratio of AD/CB[7] is close to the stoichiometric point, it is not possible to get all the data at equilibrium state (Supplementary Fig. S5). Thus only the data at equilibrium state was used for the fitting. All of the data, including the data that was not used for the fitting, matched with the fitting line very well, which offers an approach for the prediction of mechanical strength of the hydrogels.

### Tailorability of the hydrogels

**Figure 5:**
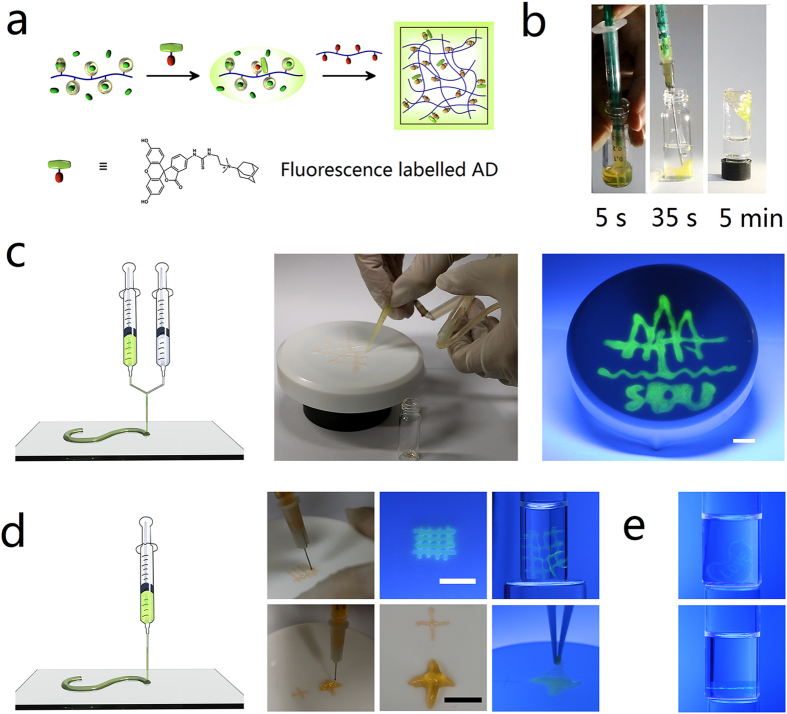
Modification and application of the hydrogels. (**a**) Fluorescence labeling of the hydrogels with FITC graft AD through host-guest interaction. (**b**) Injection of the hydrogel treated with DAH^2+^. Suction and injection of the hydrogels was performed at 5 s and 35 s after the mixing of polymer precursors. Stable hydrogels formed within 5 min. (**c**) Printing of the CB[7]-AD cross-linked hydrogel treated with DAH^2+^ by mixing the polymers solutions during printing process. Pattern of the badge of Shandong University was made with the given method and device. (**d**) Printing of CB[7]-AD cross-linked hydrogel treated with FTMA^+^ by mixing polymers solutions before printing process. Upper row demonstrates the resolution of the printing and bottom row demonstrates the printing of the hydrogel on vertical direction. The hydrogel patterns are strong enough for transfer after curing for 24 h. (**e**) Fluorescence patterning inside hydrogels. This sample is a gift made by Hao Chen for his fiancee Jing Wang for marriage proposal. All the scale bars in the figures are 1 cm.

The CB[7] pendants in the hydrogel can also be used for the modification of the hydrogels through host-guest interactions^25^,^26^. The procedure of the modification is illustrated in Fig. 5a. Fluorescence modification was done to facilitate the observations. Fluorescein isothiocyanate (FITC) was grafted onto the AD core. The functionalized AD was then attached onto the CB[7] pendent polymer through simply mixing of their solutions. The hydrogels formed with the labelled CB[7] pendent polymer and AD pendent polymer also have strong fluorescence and the labelling is quite stable during applications (*vide infra*).

### Injection and printing of the hydrogels

The control of gelation kinetics of the hydrogels was further employed for the application of injectable and printable hydrogels. FITC labelled hydrogels were used for clearer observations. The hydrogel treated with DAH^2+^ was used for the injection due to its minute scale gelation (Fig. 5b). The medium gelation kinetics offers sufficient time for a series of continuous operations, including the mixing of two polymers, suction into syringe and injection. Meanwhile, the medium gelation kinetics of the viscos pre-hydrogel mixture prevents the dilution and diffusion after its injection. Its gelation in water ensures that it is applicable under aqueous atmosphere such as *in vivo* situation with body fluid. The suitable gelation process also minimizes the possibility of the clogging of capillaries by the hydrogels.

Microextrusion printing is widely employed for hydrogel printing due to its tolerance for the viscosity of hydrogel precursors^3^. Two methods, namely mixing of the polymers solutions during printing and mixing of polymers solutions before printing were applied for the printing of hydrogels. As we do not have access to a three dimensional (3D) printer, simple alternate devices were made for feasibility verification and the nozzles were maneuvered by hand. Fast gelation kinetics of the hydrogels treated with DAH^2+^ is suitable for the method that mixing of the polymers solutions during printing with the device illustrated in Fig. 5c. Solutions of the CB[7] pendent polymer (pre-saturated with 5 equiv DAH^2+^) and AD pendent polymer were intruded into one channel from two syringe pumps through the connection of a three-way link. The solutions were mixed inside the channel and extruded from the nozzle. The mixture gelled soon after it was printed on substrate, and the pattern of the badge of Shandong University was then made. Slow gelation kinetics of the hydrogels treated with FTMA^+^ offers sufficient time for the mixing of polymer precursors, therefore the method that mixing of polymers solutions before printing is employed for these hydrogels (Fig. 5d). The CB[7] pendent polymer that pre-saturated with FTMA^+^ and the AD pendent polymer was pre-mixed by vortex mixing and loaded into a syringe pump. The mixture was aged for 2 h to give suitable mechanical strength of the hydrogels before printing. More meticulous patterns could be made through a thinner nozzle with the hydrogel strips as thin as 1 mm. Moreover, since the precursor already possess certain strength, it is applicable for the hydrogel to accumulate in the vertical direction, namely the 3D printing of the hydrogel was achieved. After 24 h, the hydrogel patterns are strong enough for removing from the pristine substrate and transportation. The hydrogel patterns stay intact even after preserved in water for months and they still have strong fluorescence. As controllable spatial complexity of hydrogels is also important for their application, the patterning inside hydrogel was also demonstrated through the combination of casting and printing of the non-labeled and labeled CB[7]-AD cross-linked hydrogels (Fig. 5e). The pattern inside the hydrogel did not blur even after months, showing that the extra slow relaxation of CB[7]-AD endue good stability of the supramolecular modification which even comparable to covalent modification.

## Discussion

The method that control gelation kinetics of supramolecular hydrogels with competing guest molecules was addressed based on the study of the CB[7]-AD cross-linked hydrogels. The gelation of the CB[7] pendent polymer and AD pendent polymer was decelerated through pre-saturate the CB[7] pendent polymer with competing guest molecules. Additional disassociation process of the competing guests from CB[7] cavity is attributed to the deceleration. It is no doubt that the disassociation dynamics of the competing guests affects the disassociation process. Meanwhile, the competing re-binding of the competing guests, which determined by the association dynamics and concentration of the competing guests, also have an effect on the disassociation process. Hence, two factors, namely sort and concentration of the competing guest molecules, determine the gelation kinetics. There is a fundamental difference that distinguishes the hydrogels from the previously reported supramolecular hydrogels. Here we try to use the dynamic process of supramolecular interactions whilst the previous reported ones usually discuss the transition of supramolecular interactions between various thermodynamics states^35^,^36^.

Although it is a known principle that the host-guest interaction could be decelerated by competing guests. However, in order to make the theory practical, the competing guest molecules should have sufficiently slow dissociation rate to guarantee the gelation kinetics is effectively decelerated. The respective binding affinities of MV^2+^, DAH^2+^ and FTMA^+^ with CB[7] in pure water are (9.5 ± 1.1) × 10^7^, (2.1 ± 0.4) × 10^9^ and (4.1 ± 1.0) × 10^12^ M^−1^ 37,38, therefore the lifetime of their host-guest complexes with CB[7] are considerable even if their association rate is close to diffusion-controlled rate constant (∼10^9^ M^−1^ s^−1^)^23^,^39^. The equilibrium association constant of the supramolecular crosslinks (here is CB[7]-AD) must be even larger, or the crosslinks cannot form.

The properties (e.g. mechanical properties) of supramolecular polymeric hydrogels are largely dependent on the non-covalent interactions that agglomerate them together^40^,^41^,^42^,^43^,^44^. Therefore the high equilibrium association constants and slow dissociation dynamics of the CB[7]-AD interactions will undoubtedly reflect on the properties of the hydrogels. In comparison with most other supramolecular hydrogels, the CB[7]-AD cross-linked hydrogels demonstrate high mechanical strength with very low solid content and crosslink density. Moreover, the slow dissociation dynamics guarantee them stay intact even under considerable applied force, thus the hydrogels demonstrate high elasticity and shape-persistence. Furthermore, the hydrogels also display good flexibility, which may be attributed to their low crosslink density. To some extent, the hydrogels behave more like covalently cross-linked hydrogels rather than supramolecular hydrogels due to the special CB[7]-AD interaction.

The gelation mechanism is useful for hydrogel process and varies hydrogel based applications such as hydrogel injection and printing. Moreover, the supramolecular inherence endues the hydrogels with good tailorability. Based on these features, this gelation mechanism is hopefully a new tool for the development of hydrogels with proper temporal and spatial complexity which is a trend of hydrogel technology^1^,^17^,^45^.

## Methods

### Instrumentation

NMR tests were performed using two NMR spectrometers. A Bruker Avance 400 NMR spectrometer was used for the characterization of synthetic compounds and polymers. And an Agilent 600 MHz NMR spectrometer was employed for the monitoring of gelation process of the hydrogels in order to achieve a good clarity. All of the NMR tests were performed at 298 K. Mass spectrometry was tested on an Agilent 6520 accurate mass Q-TOF. Gel permeation chromatography (GPC) was carried out in water utilizing a Shimadzu CBM-20A system controller and RID-10A refractive index detector with Shodex OHpak SB columns. Samples were filtered with MILLEX-GV 0.22 μm filter before loading.

Rheological experiments were measured using a TA Discovery Hybrid Rheometer fitted with a 40 mm 4° cone geometry and a standard peltier plate. The gelation processes of the hydrogels were recorded at a frequency of 1 Hz and a strain of 0.02. The strain-dependent oscillatory rheology and frequency-dependent oscillatory rheology of the hydrogels were tested at a frequency of 1 Hz and a strain of 0.02, respectively. All of the data was obtained at 298 K.

Compression tests of the hydrogels were performed using a TA DMA Q800 dynamic mechanical analyzer equipped with compression clamp geometry at 298 K. The hydrogels were casted into tablet shape with diameter of 12.4 mm and height of 3.5 mm. All the samples were aged for over 10 days which left enough time for the gelation process. The compression rate of samples is 10%/min for compression experiments and 30%/min for compression-decompression cycles.

### CB[7] pendent polymer

The CB[7] pendent polymer used in this study was synthesized based on an established method of our group^30^. Generally, A CB[7] based monomer, 4-vinylbenzyloxy CB[7], was synthesized through firstly oxidize CB[7] to obtain mono-hydroxy CB[7] before graft 4-vinylbenzyl group onto the hydroxyl group. Then the CB[7] monomer was copolymerized with *N,N*-dimethylacrylamide to yield the desired CB[7] pendent polymer. 3,3′-(Octane-1,8-diyl)-bis-(1-ethyl-imidazolium) dibromide (C_8_bim) which is used to enhance the solubility of the CB[7] monomer was added before the polymerization and it was removed from the polymer through dialysis the polymer against CB[7] solution and pure water. The molecular weight and composition of the CB[7] pendent copolymer were determined by GPC and NMR methods respectively. Its M_n_ is 5.49 × 10^−5^ g mol^−1^ with M_w_/M_n_ = 3.77 and the content of CB[7] units is 0.74 mol%.

### AD pendent polymer

An adamantane based acrylamide monomer (compound **7** in supplementary information, 0.39 mmol) was dissolved in 25 mL deionized water with *N,N*-dimethyl acrylamide (1.26 g, 0.013 mol). The solution was degassed under vacuum and purged with nitrogen for three cycles. (NH_4_)_2_S_2_O_8_ solution contains 0.0050 g (2.2 × 10^−5^ mol) solute was injected into the monomer solution followed by NaHSO_3_ solution contains 0.0018 g (1.8 × 10^−5^ mol) solute. The polymerization was conducted at 33 °C for 12 hours. The polymer solution was dialyzed in deionized water using semi-permeable membrane (MWCO: 8000 – 14000) for three days to remove unreacted monomers and impurities. Then the polymer solution was further concentrated as a stock solution and ready for use (total weight of the polymer solution is 11.63 g with concentration of 9.74 w/w%, yield of the copolymer is 80.7%). The copolymer was tested with M_n_ 3.93 × 10^−5^ g mol^−1^ and M_w_/M_n_ = 2.69. The content of AD pendants were identified by ^1^H NMR of 2.54 mol%.

### Preparation of the CB[7]-AD cross-linked hydrogels with controlled gelation kinetics by using competing guest molecules

The stock solution of CB[7] pendent polymer was firstly mixed with competing guest molecules and diluted to 2 wt%. The sort and the concentration of the competing guest molecules varied based on experiments. Usually MV^2+^, DAH^2+^ and FTMA^+^ with amount range from 1 to 14 equiv *versus* CB[7] moiety were utilized. Then the guest molecule pre-saturated CB[7] pendent polymer was mixed with 2 wt% AD pendent polymer under vortex mixing (1200 rpm) which generated hydrogels with 2 wt% solid content. The mixing ratio of the two polymer solutions is usually 3.4:1 (v/v) which is corresponding to the 1:1 stoichiometric point of CB[7]/AD pair (unless otherwise noted). The gelation time varies from seconds to hours depending on the sort and concentration of competing guest molecules.

### FITC labelling of the CB[7] pendent polymer

The stock solution of CB[7] pendent polymer was firstly mixed with competing guest molecules. The sort and the concentration of the competing guest molecules varied based on certain experiments. Here DAH^2+^ and FTMA^+^ with 3 or 5 equiv *versus* CB[7] moiety were utilized. Then the solution of FITC labelled AD (the preparation procedure is available in supplementary information, 0.1 equiv *versus* CB[7] moiety) was added and the solution was mixed under vortex mixing (1200 rpm). The solution then diluted with deionized water to 2 wt%. After stand for 1 h, the FITC labelled CB[7] pendent polymer solution is ready for use.

### Injection of the CB[7]-AD cross-linked hydrogels

The FITC labelled CB[7] pendent polymer (1.0 mL, 2 wt%, 5 equiv DAH^2+^) was mixed with the AD pendent polymer (0.368 mL, 2 wt%) through vortex mixing (1200 rpm) for 3 s. The stoichiometry between CB[7] and AD pendants is calculated as 1:0.8. The mixed solution was sucked into a syringe and ready for injection. The solution was injected into water through a 22 gauge needle and it gelled after several minutes with a clear interface in water.

### Printing of the CB[7]-AD cross-linked hydrogels by mixing of the polymers solutions during printing

The FITC labelled CB[7] pendent polymer (2 wt%, 5 equiv DAH^2+^) and AD pendent polymer (0.5 wt%) was loaded in two separate syringes. These syringes were loaded into one syringe pump with flow rate set as 0.94 mL/min for each syringe. The solutions were intruded into one channel (diameter is 2 mm with 30 cm length) from two syringes through the connection of a three-way link. Thus the two polymers were mixed for 30 s before extruding. A 200 μL tip of pipettes was modified as a nozzle. The nozzle was maneuvered by hand following the solutions was extruded out of the nozzle, and patterns were then made on Teflon surface.

### Printing of the CB[7]-AD cross-linked hydrogels by mixing of the polymers solutions before printing

The FITC labelled CB[7] pendent polymer (0.5 mL, 2 wt%, 3 equiv FITC^+^) was mixed with the AD pendent polymer (0.184 mL, 2 wt%) through vortex mixing (1200 rpm) for 10 s. The mixed solution was loaded into a syringe and aged for 2 h. The mixed solution gained certain strength based on the gelation process given in supplementary Fig. S2. After that the mixed solution was extruded out through a 26 gauge needle which worked as the nozzle. The nozzle was maneuvered by hand and patterns were made on Teflon surface.

## Additional Information

**How to cite this article**: Chen, H. *et al.* Controlled gelation kinetics of cucurbit[7]uril-adamantane cross-linked supramolecular hydrogels with competing guest molecules. *Sci. Rep.* **6**, 20722; doi: 10.1038/srep20722 (2016).

## Supplementary Material

Supplementary Information

## Figures and Tables

**Figure 1:**
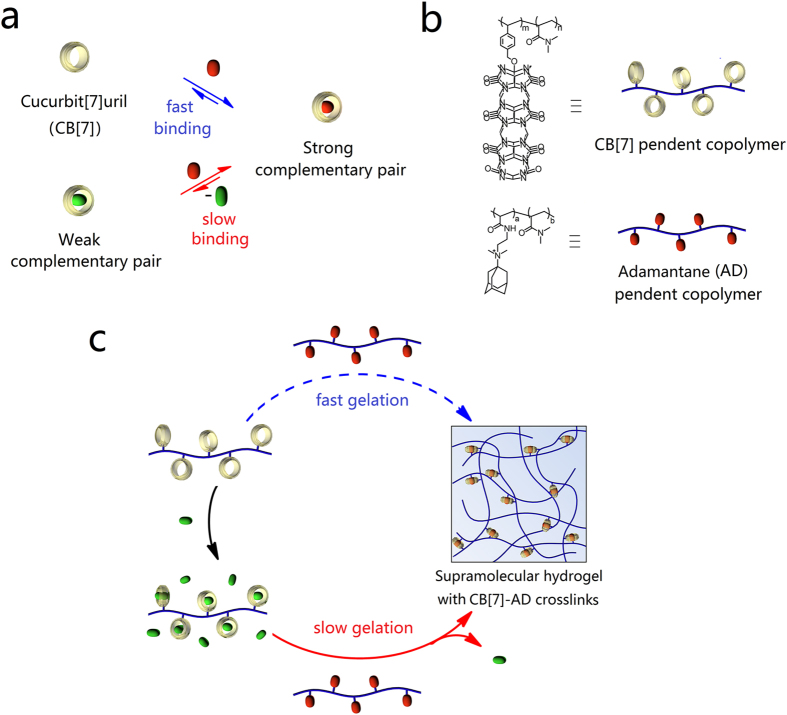
Schematic of control gelation kinetics of the CB[7]-AD cross-linked hydrogels with competing guest molecules. (**a**) The formation of a strong CB[7]-AD complementary pair is fast. This process can be impeded by pre-saturate CB[7] with a weak competing guest molecule before its interaction with the AD core. (**b**) Chemical structures of the CB[7] pendent polymer and AD pendent polymer. (**c**) The gelation kinetics of the CB[7]-AD cross-linked hydrogels can also be decelerated by using competing guest molecules.

## References

[b1] Li Y., Rodrigues J. & Tomás H. Injectable and biodegradable hydrogels: gelation, biodegradation and biomedical applications. Chem. Soc. Rev. 41, 2193–2221 (2012). 22116474 10.1039/c1cs15203c

[b2] Moon H. J., Ko D. Y., Park M. H., Joo M. K. & Jeong B. Temperature-responsive compounds as *in situ* gelling biomedical materials. Chem. Soc. Rev. 41, 4860–4883 (2012). 22688789 10.1039/c2cs35078e

[b3] Murphy S. V. & Atala A. 3D bioprinting of tissues and organs. Nat. Biotechnol. 32, 773–785 (2014). 25093879 10.1038/nbt.2958

[b4] Joo M. K., Park M. H., Choi B., G. & Jeong, B. Reverse thermogelling biodegradable polymer aqueous solutions. J. Mater. Chem. 19, 5891–5905 (2009).

[b5] Chen H. *et al.* Aggregation and thermal gelation of *N*-isopropylacrylamide based cucurbit[7]uril side-chain polypseudorotaxanes with low pseudorotaxane content. RSC Adv. 5, 20684–20690 (2015).

[b6] Pataky K. *et al.* Microdrop printing of hydrogel bioinks into 3D tissue-like geometries. Adv. Mater. 24, 391–396 (2012). 22161949 10.1002/adma.201102800

[b7] Zhao L., Weir M. D. & Xu H. H. An injectable calcium phosphate-alginate hydrogel-umbilical cord mesenchymal stem cell paste for bone tissue engineering. Biomaterials. 31, 6502–6510 (2010). 20570346 10.1016/j.biomaterials.2010.05.017 PMC3001248

[b8] Chiu Y. L. *et al.* pH-Triggered injectable hydrogels prepared from aqueous *N*-palmitoyl chitosan: *in vitro* characteristics and *in vivo* biocompatibility. Biomaterials 30, 4877–4888 (2009). 19527916 10.1016/j.biomaterials.2009.05.052

[b9] Wang Q., Wang J. X., Lu Q. H., Detamore M. S. & Berkland C. Injectable PLGA based colloidal gels for zero-order dexamethasone release in cranial defects. Biomaterials 31, 4980–4986 (2010). 20303585 10.1016/j.biomaterials.2010.02.052 PMC2856787

[b10] Shi J. *et al.* Schiff based injectable hydrogel for *in situ* pH-triggered delivery of doxorubicin for breast tumor treatment. Polym. Chem. 5, 61s80–6189 (2014).

[b11] Purcell B. P. *et al.* Injectable and bioresponsive hydrogels for on-demand matrix metalloproteinase inhibition. Nat. Mater. 13, 653–661 (2014). 24681647 10.1038/nmat3922 PMC4031269

[b12] Yang F. *et al.* Injectable and redox-responsive hydrogel with adaptive degradation rate for bone regeneration. J. Mater. Chem. B. 2, 295–304 (2014). 10.1039/c3tb21103g 32261508

[b13] Xu G. *et al.* Injectable biodegradable hybrid hydrogels based on thiolated collagen and oligo(acryloyl carbonate)–poly(ethylene glycol)–oligo(acryloyl carbonate) copolymer for functional cardiac regeneration. Acta Biomater. 15, 55–64 (2015). 25545323 10.1016/j.actbio.2014.12.016

[b14] Shepherd J. N. H. 3D Microperiodic hydrogel scaffolds for robust neuronal cultures. Adv. Funct. Mater. 21, 47–54 (2011). 21709750 10.1002/adfm.201001746 PMC3120232

[b15] Billiet T., Gevaert E., De Schryver T., Cornelissen M. & Dubruel P. The 3D printing of gelatin methacrylamide cell-laden tissue-engineered constructs with high cell viability. Biomaterials 35, 49–62 (2014). 24112804 10.1016/j.biomaterials.2013.09.078

[b16] Gou M. *et al.* Bio-inspired detoxification using 3D-printed hydrogel nanocomposites. Nat. Commun. 5, 3774 (2014). 24805923 10.1038/ncomms4774 PMC4024742

[b17] Ko D. Y., Shinde U. P., Yeon B. & Jeong B. Recent progress of *in situ* formed gels for biomedical applications. Prog. Polym. Sci. 38, 672–701 (2013).

[b18] Jin R. *et al.* Synthesis and characterization of hyaluronic acid–poly(ethylene glycol) hydrogels *via* Michael addition: An injectable biomaterial for cartilage repair. Acta Biomater. 6, 1968–1977 (2010). 20025999 10.1016/j.actbio.2009.12.024

[b19] Tan H., Chu C. R., Payne K. A. & Marra K. G. Injectable *in situ* forming biodegradable chitosan–hyaluronic acid based hydrogels for cartilage tissue engineering. Biomaterials 30, 2499–2506 (2009). 19167750 10.1016/j.biomaterials.2008.12.080 PMC2676686

[b20] Márquez C., Hudgins, R. R. & Nau W. M. Mechanism of host-guest complexation by cucurbituril. J. Am. Chem. Soc. 126, 5806–5816 (2004). 15125673 10.1021/ja0319846

[b21] Tang H. *et al.* Guest binding dynamics with cucurbit[7]uril in the presence of cations. J. Am. Chem. Soc. 133, 20623–20633 (2011). 22073977 10.1021/ja209266x

[b22] Mukhopadhyay P., Zavalij P. Y. & Isaacs L. High fidelity kinetic self-sorting in multi-component systems based on guests with multiple binding epitopes. J. Am. Chem. Soc. 128, 14093–14102 (2006). 17061892 10.1021/ja063390j PMC2529227

[b23] Tootoonchi M. H. Yi S. & Kaifer A. E. Detection of isomeric microscopic host−guest complexes. A time-evolving cucurbit[7]uril complex. J. Am. Chem. Soc. 135, 10804–10809 (2013). 23795622 10.1021/ja404797y

[b24] Moghaddam S. *et al.* New ultrahigh affinity host-guest complexes of cucurbit[7]uril with bicyclo[2.2.2]octane and adamantane guests: thermodynamic analysis and evaluation of M2 affinity calculations. J. Am. Chem. Soc. 133, 3570–3581 (2011). 21341773 10.1021/ja109904u PMC3065999

[b25] Jung H. *et al.* 3D tissue engineered supramolecular hydrogels for controlled chondrogenesis of human mesenchymal stem cells. Biomacromolecules 15, 707–714 (2014). 24605794 10.1021/bm401123m

[b26] Park K. M. *et al.* *In situ* supramolecular assembly and modular modification of hyaluronic acid hydrogels for 3D cellular engineering. ACS Nano 6, 2960–2968 (2012). 22404424 10.1021/nn204123p

[b27] Lee J. W., Samal S., Selvapalam N., Kim H.-J. & Kim K. Cucurbituril homologues and derivatives: new opportunities in supramolecular chemistry. Acc. Chem. Res. 36, 621–630 (2003). 12924959 10.1021/ar020254k

[b28] Lagona J., Mukhopadhyay P., Chakrabarti S. & Isaacs L. The cucurbit[n]uril family. Angew. Chem. Int. Ed. 44, 4844–4870 (2005). 10.1002/anie.200460675 16052668

[b29] Masson E., Ling X., Joseph R., Kyeremeh-Mensah L. & Lu X. Cucurbituril chemistry: a tale of supramolecular success. RSC Adv. 2, 1213–1247 (2012).

[b30] Chen H., Ma H. & Tan Y. Synthesis of linear cucurbit[7]uril pendent copolymers through radical polymerization: Polymers with ultra-high binding affinity. J. Polym. Sci., Part A: Polym. Chem. 53, 1748–1752 (2015).

[b31] Mock W. L. & Shih N.-Y. Host-Guest Binding Capacity of Cucurbituril. J. Org. Chem. 48, 3618–3619 (1983).

[b32] Jia Y.-G. & Zhu X. X. Self-healing supramolecular hydrogel made of polymers bearing cholic acid and β-cyclodextrin pendants, Chem. Mater. 27, 387–393 (2015).

[b33] McKee J. R. *et al.* Healable, stable and stiff hydrogels: combining conflicting properties using dynamic and selective three-component recognition with reinforcing cellulose nanorods. Adv. Funct. Mater. 24, 2706–2713 (2014).

[b34] Xu D. & Craig S. L. Strain hardening and strain softening of reversibly cross-linked supramolecular polymer networks, Macromolecules 44, 7478–7488 (2011). 22043083 10.1021/ma201386t PMC3203206

[b35] Takashima Y. *et al.* Expansion–contraction of photoresponsive artificial muscle regulated by host–guest interactions, Nat. Commun. 3, 1270 (2012). 23232400 10.1038/ncomms2280 PMC3535346

[b36] Nakahata M., Takashima Y., Yamaguchi H. & Harada A. Redox-responsive self-healing materials formed from host−guest polymers, Nat. Commun. 2, 511 (2011). 22027591 10.1038/ncomms1521 PMC3207205

[b37] Chen H., Yang H., Xu W. & Tan Y. A supramolecular switch based on three binding states of a pyrene derivate: a reversible three-state switch with only two stimuli. RSC Adv. 3, 13311–13317 (2013).

[b38] Rekharsky M. V. *et al.* A synthetic host-guest system achieves avidin-biotin affinity by overcoming enthalpy– entropy compensation. Proc. Natl Acad. Sci. USA 104, 20737–20742 (2007). 18093926 10.1073/pnas.0706407105 PMC2410071

[b39] Montalti M., Credi A., Prodi L. & Gandolfi M. T. Handbook of Photochemistry [3rd ed.] (CRC Press, Boca Raton, 2006).

[b40] Appel E. A., del Barrio J., Loh X. J. & Scherman O. A. Supramolecular polymeric hydrogels. Chem. Soc. Rev. 41, 6195–6214 (2012). 22890548 10.1039/c2cs35264h

[b41] Wei Z. *et al.* Self-healing gels based on constitutional dynamic chemistry and their potential applications. Chem. Soc. Rev. 43, 8114–8131 (2014). 25144925 10.1039/c4cs00219a

[b42] Aida T., Meijer E. W. & Stupp S. I. Functional Supramolecular Polymers. Science 335, 813–817 (2012). 22344437 10.1126/science.1205962 PMC3291483

[b43] Meijer E. W. & de Greef T. F. A. Supramolecular polymers. Nature 453, 171–173 (2008). 18464733 10.1038/453171a

[b44] Seiffert S. & Sprakel J. Physical chemistry of supramolecular polymer networks. Chem. Soc. Rev. 41, 909–930 (2012). 21909565 10.1039/c1cs15191f

[b45] Freudenberg U. A star-PEG–heparin hydrogel platform to aid cell replacement therapies for neurodegenerative diseases. Biomaterials 30, 5049–5060 (2009). 19560816 10.1016/j.biomaterials.2009.06.002

