# Supplementary material for: Controlled gelation kinetics of cucurbit[7]uril-adamantane cross-linked supramolecular hydrogels with competing guest molecules
Source: Sci Rep. 2016 Feb 5;6:20722. doi: 10.1038/srep20722 (PMC4742902; doi:10.1038/srep20722)
Supplement: Supplementary Information [file srep20722-s1.pdf]

Controlled gelation kinetics of cucurbit[7]uril-adamantane cross-linked supramolecular hydrogels with competing guest molecules

Hao Chen[1,2], Shengzhen Hou[1,2], Haili Ma[1,2], Xu Li[3]* & Yebang Tan[1,2]*

[1]School of Chemistry and Chemical Engineering, Shandong University, Jinan 250100, People's Republic of China.

[2]The Key Laboratory of Special Functional Aggregated Materials, Ministry of Education, Shandong University, Jinan 250100, People's Republic of China.

[3]Institute of Materials Research and Engineering, 3 Research Link, Singapore 117602.

Correspondence and requests for materials should be addressed to Y.T. (email: ybtan@sdu.edu.cn) and X.L. (email: x-li@imre.a-star.edu.sg).

Materials

Amantadine hydrochloride, di-*tert*-butyl dicarbonate, 18-crown-6, 2-bromoethylamine hydrobromide, *N,N*-diisopropyl ethylamine, acryloyl chloride, anhydrous dimethyl formamide and *N,N*-dimethyl acrylamide were purchased from J&K Scientific. 1,6-Diaminohexane dihydrochloride (DAH^{2+}) was obtained from Tokyo Chemical Industry Co., Ltd.. Ferrocenyl trimethyl ammonium iodide ($FTMA^{+}$), fluorescein isothiocyanate (FITC) and semi-permeable membrane were received from Sigma-Aldrich. Dichloromethane, potassium carbonate, acetonitrile, mineral acid and ethyl ether were purchased from Sinopharm Chemical Reagent Co., Ltd.. All of the chemicals were used directly without further purification. Dimethyl viologen (MV^{2+}) was synthesized based on our previous report[1].

Synthesis procedures of AD based acrylamide monomer and AD pendent copolymer

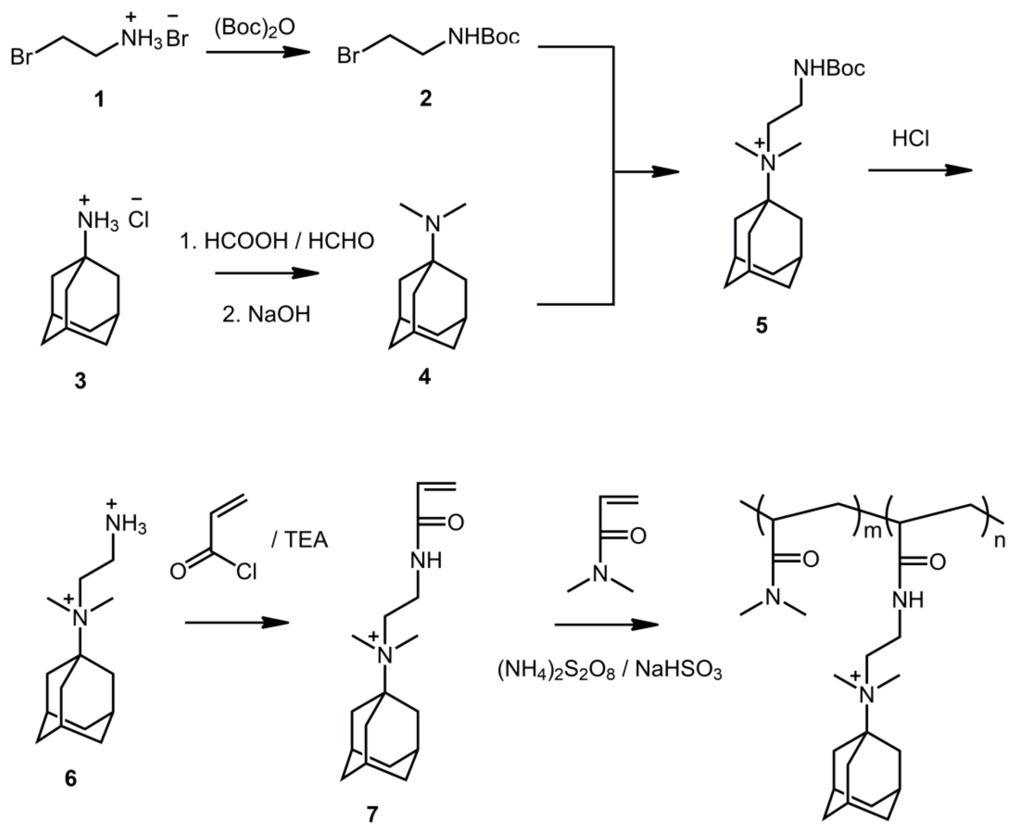

Synthesis of compound 2: Di-*tert*-butyl dicarbonate ($(Boc)_2O$, 12.4 g, 0.059 mol) was dissolved in 80 mL anhydrous dichloromethane. Potassium carbonate (13.8 g, 0.1 mol) and 18-crown-6 (0.2 g, 0.76 mmol) was then added into the solution. The suspension was chilled in ice bath under stirring. After the suspension was cooled down, 2-bromoethylamine hydrochloride (compound **1**) (10 g, 0.049 mol) was added into the suspension and reacted for 12 h. The suspension was filtrated after the reaction. The filtrate was washed with water for 3 times, desiccated with sodium carbonate and evaporated to obtain the final product as clear liquid (10.3 g, 94 %). ^{1}H NMR (400 MHz, $CDCl_3$) δ 5.12 (s, 1H), 3.52 (t, 2H, J = 5.4 Hz), 3.45 (t, 2H, J = 5.4 Hz), 1.45 (s, 9H). ^{13}C NMR (100 MHz, D_2O) δ 155.64, 79.41, 42.29, 32.20, 28.24.

Synthesis of compound 4: The procedure has been reported in a previous work[2].

Synthesis of compound 5: Compound **2** (1.2 g, 0.0054 mol) and compound **4** (1 g, 0.0056 mol) was dissolved in a mixed solvent of 25 mL acetonitrile and 5 mL water. Potassium carbonate (1.5 g) was added into the solution to keep a basic atmosphere. The reaction was conducted at 65 °C for 12 h and evaporated to obtain white solid. The solid was then extracted with acetonitrile for three times (20 mL, 10 mL, 10 mL) and which was concentrated to 10 mL. The solution was then precipitated with ethyl ether to obtain compound **5** as white solid (0.15 g, 6.9 %). ^{1}H NMR (400 MHz, D_2O) δ 3.56 (t, J = 6.9 Hz, 2H), 3.31 (t, J = 6.9 Hz, 2H), 2.93 (s, 6H), 2.30 (s, 3H), 2.07 (d,

6H), 1.71 (d, J = 12.4 Hz, 3H), 1.63 (d, J = 12.7 Hz, 3H), 1.42 (s, 6H). ^{13}C NMR (100 MHz, D_2O) δ 157.81, 81.83, 69.83, 60.95, 55.76, 41.99, 34.65, 34.24, 30.37, 27.72. HRMS: $[M]^+$ calcd for $C_{19}H_{35}N_2O_2^+$, 323.2699; found 323.2830.

Synthesis of compound 6: Compound **5** (0.31 g, 0.77 mmol) was dissolved in 10 mL 1 M HCl aqueous solution and stirred for 1 h. The solution was evaporated to obtain compound **6** (0.245 g, 94 %). ^{1}H NMR (400 MHz, D_2O) δ 3.47 (s, 4H), 2.88 (s, 6H), 2.24 (s, 3H), 2.00 (d, J = 2.7 Hz, 6H), 1.62 (d, J = 12.6 Hz, 3H), 1.55 (d, J = 12.4 Hz, 3H). ^{13}C NMR (100 MHz, D_2O) δ 76.47, 55.72, 43.57, 34.62, 34.20, 30.35, 27.68. HRMS: $[M + Br^-]^+$ calcd for $C_{14}H_{28}N_2Br^+$, 303.1436; found 303.1240.

Synthesis of compound 7: Compound **6** (0.34 g, 1 mmol) was dissolved in a mixed solution of 1 mL *N,N*-diisopropyl ethylamine and 12 mL dimethyl formamide. Acryloyl chloride (160 µL, 2 mmol) was added at 0 °C and reacted for 10 h. The solution was precipitated with ethyl ether after the reaction. Compound **7** was obtained as a mixture of it with *N,N*-diisopropyl ethylamine hydrochloride (total weight of the precipitate is 0.72 g). The molar ratio of the mixture was identified as 1:2.65 for compound **7** and *N,N*-diisopropyl ethylamine hydrochloride based on ^{1}H NMR result. Since *N,N*-diisopropyl ethylamine hydrochloride is inert for polymerization, the mixture was directly used for polymerization without further purification. ^{1}H NMR (400 MHz, D_2O) δ 6.25 (d, J = 9.3 Hz, 1H), 6.24 (d, J = 2.8 Hz, 1H), 5.80 (dd, J = 9.0, 2.5 Hz, 1H), 3.77 (t, J = 7.1 Hz, 2H), 3.39 (t, J = 7.1 Hz, 2H), 2.31 (s, 3H), 2.07 (d, J = 2.3 Hz, 6H), 1.71 (d, J = 12.5 Hz, 3H), 1.64 (d, J = 12.4 Hz, 3H). ^{13}C NMR (100 MHz, D_2O) δ 169.30, 129.88, 128.62, 77.04, 55.48, 43.93, 34.98, 34.58, 34.30, 30.73. HRMS: $[M]^+$ calcd for $C_{17}H_{29}N_2O^+$, 277.2280; found 277.2301.

Synthesis of FITC labeled AD

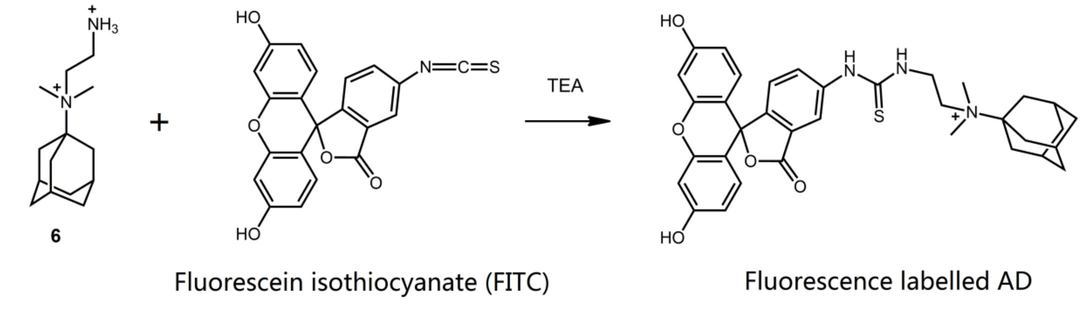

Compound **6** (0.0034 g, 0.01 mmol) was dissolved in 1 mL anhydrous acetonitrile with triethylamine (TEA, 2 µL, 0.014 mmol), then fluorescein isothiocyanate (FITC, 0.0043 g, 0.011 mmol) was added and stirred for 48 h at room temperature. After the reaction, the mixture was desiccated under argon flow and dissolved in 10 mL deionized water. The aqueous solution of FITC labelled AD (calculated as 1 mM) was directly used without further purification.

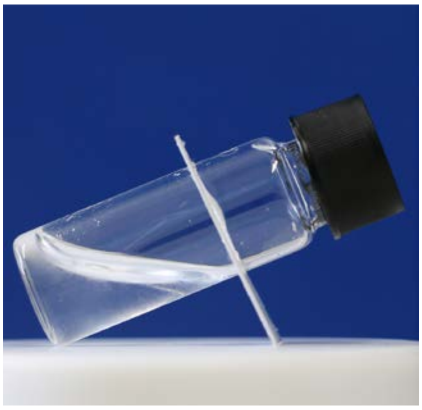

Figure S1 Directly mixing of the CB[7] pendent polymer with the AD pendent polymer by vortex. Aggregates rather than integral hydrogel formed due to the fast interaction between CB[7] and AD pendants.

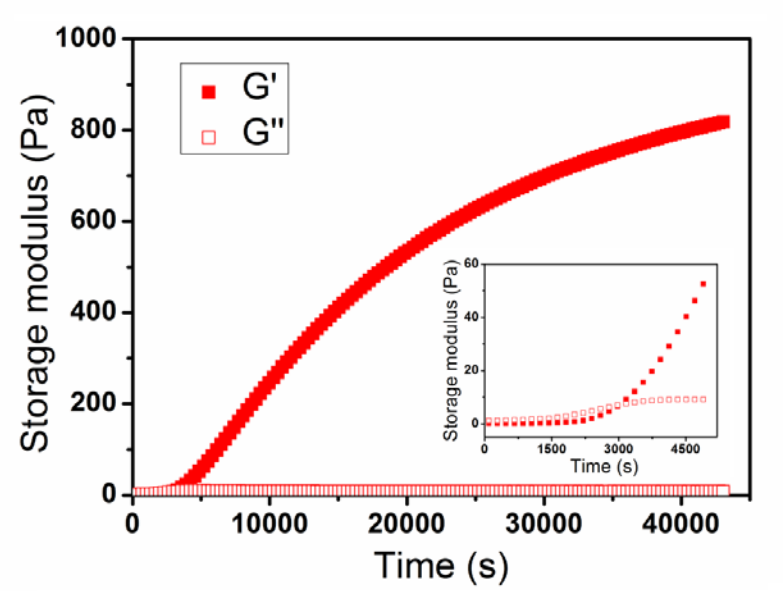

Figure S2 Gelation process of CB[7]-AD cross-linked hydrogel treated with 5 equiv $FTMA^+$. The experiment was performed with solid content of 2 wt% with CB[7]-AD at the stoichiometric point (1:1 in mole).

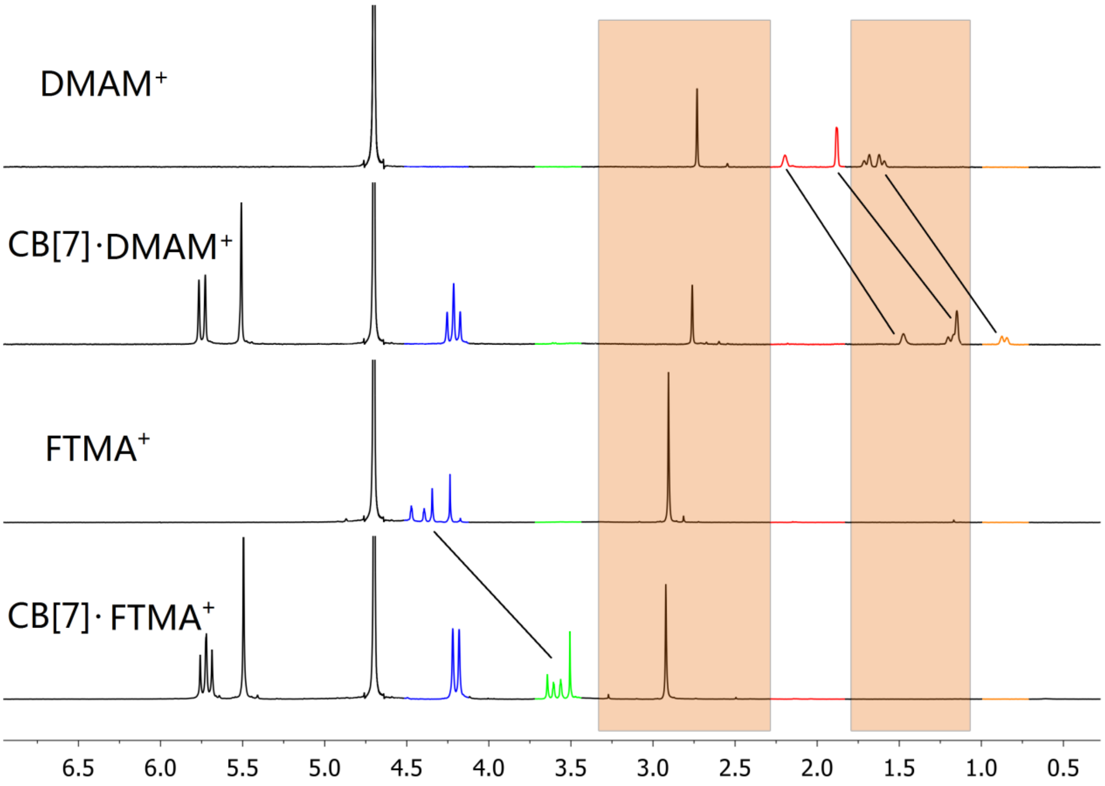

Figure S3 Supramolecular interactions of CB[7] with $FTMA^+$ and *N*,*N*-dimethylamantadine hydrochloride ($DMAM^+$). Blue for signals corresponding to protons of free $FTMA^+$, green for protons of bounded $FTMA^+$, red for protons of unbounded $DMAM^+$ and orange for protons of bounded $DMAM^+$. Patches indicate the potential overlap of signals of poly(*N*,*N*-dimethyl acrylamide) backbone. Generally, the proton signals of guest molecules display significant up-field shift when the guests are encapsulated inside CB[7] cavity.

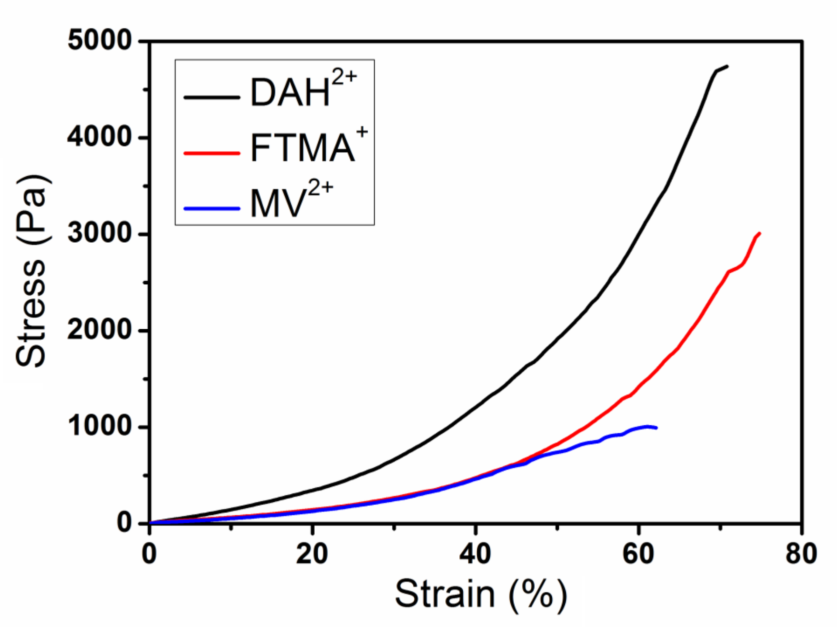

Figure S4 Compression experiments of the hydrogel treated with different competing guest molecules. Although in theory the hydrogels should have same mechanical properties as they share same CB[7]-AD crosslinks. However, things are complex in reality. The gelation of MV^{2+} treated hydrogel is too fast and its structure is not in good homogeneity. And for hydrogels that treated with $FTMA^{+}$, the gelation is too slow for thorough equilibrium. The hydrogel that treated with DAH^{2+} balances the two defects and demonstrates highest mechanical strength. The experiment was performed with solid content of 2 wt% with CB[7]-AD at the stoichiometric point (1:1 in mole).

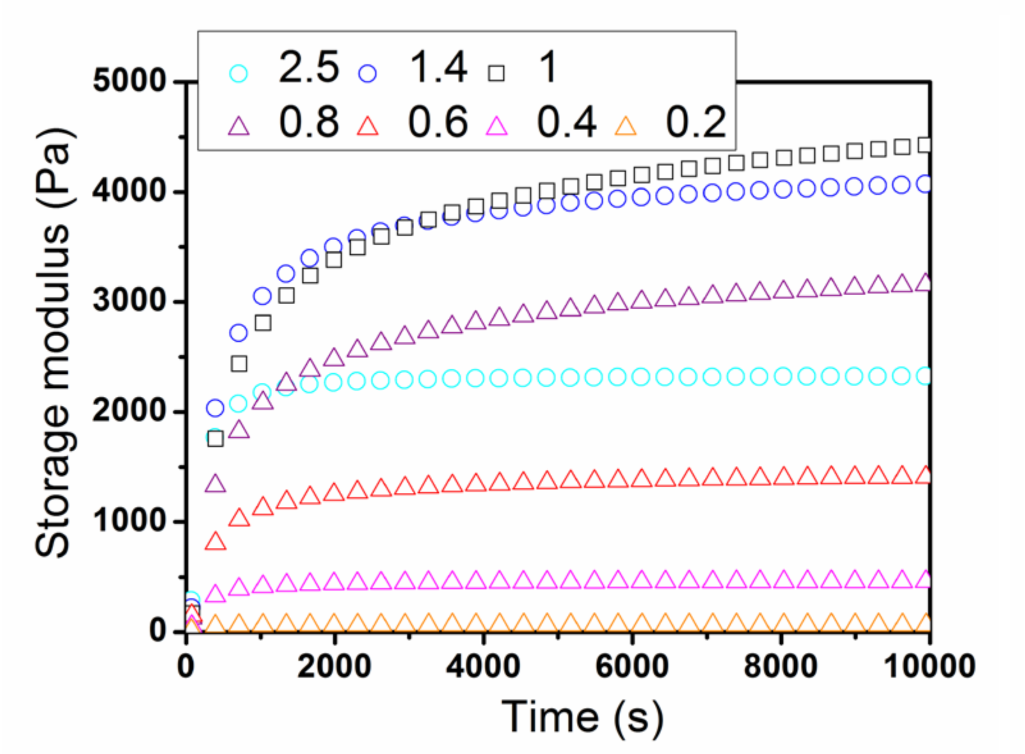

Figure S5 Gelation processes of CB[7]-AD cross-linked hydrogel with various AD/CB[7] ratio monitored by oscillatory rheological experiments. The experiment was performed with hydrogels with 2 wt% solid content which treated with 3 equiv DAH^{2+}.

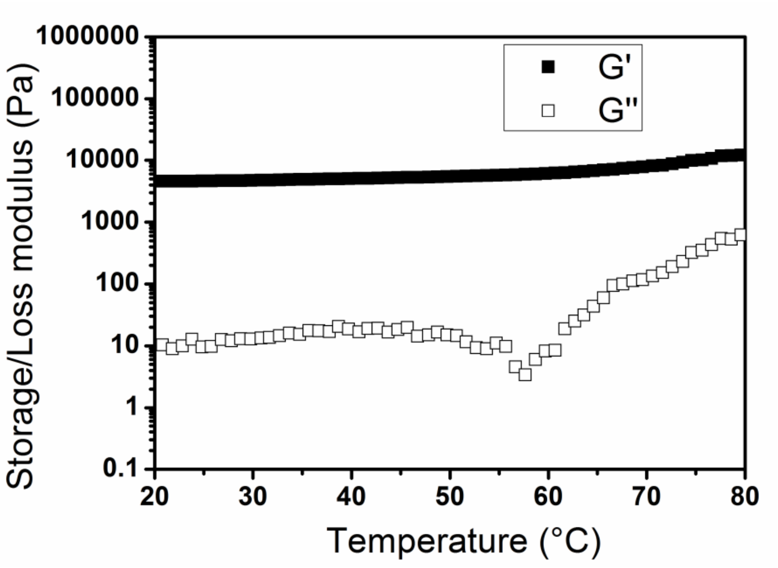

Figure S6 Oscillation temperature sweep of the CB[7]-AD cross-linked hydrogel with CB[7]/AD at the stoichiometric point. The hydrogel is stable under a wide range of temperature.

Supplementary References

1. Chen, H., Yang, H., Xu, W. & Tan, Y. A supramolecular switch based on three binding states of a pyrene derivate: a reversible three-state switch with only two stimuli. *RSC Adv.* **3,** 13311–13317 (2013).
2. Chen, H. *et al.* Aggregation and thermal gelation of *N*-isopropylacrylamide based cucurbit[7]uril side-chain polypseudorotaxanes with low pseudorotaxane content. *RSC Adv.* **5,** 20684-20690 (2015).
